# Surface States Enhanced Dynamic Schottky Diode Generator with Extremely High Power Density Over 1000 W m^−2^

**DOI:** 10.1002/advs.201901925

**Published:** 2019-10-25

**Authors:** Shisheng Lin, Runjiang Shen, Tianyi Yao, Yanghua Lu, Sirui Feng, Zhenzhen Hao, Haonan Zheng, Yanfei Yan, Erping Li

**Affiliations:** ^1^ College of Microelectronics College of Information Science and Electronic Engineering Zhejiang University Hangzhou 310027 China; ^2^ State Key Laboratory of Modern Optical Instrumentation Zhejiang University Hangzhou 310027 China; ^3^ Key Laboratory of Micro‐Nano Electronics and Smart System of Zhejiang Province College of Information Science and Electronic Engineering Zhejiang University Hangzhou 310027 China

**Keywords:** dynamic Schottky generators, high current density, high power density, rebounding centers, surface states

## Abstract

The overloaded energy cost has become the main concern of the now fast developing society, which make novel energy devices with high power density of critical importance to the sustainable development of human society. Herein, a dynamic Schottky diode based generator with ultrahigh power density of 1262.0 W m^−2^ for sliding Fe tip on rough p‐type silicon is reported. Intriguingly, the increased surface states after rough treatment lead to an extremely enhanced current density up to 2.7 × 10^5^ A m^−2^, as the charged surface states can effectively accelerate the carriers through large atomic electric field, while the reflecting directions are regulated by the built‐in electric field of the Schottky barrier. This research provides an open avenue for utilizing the surface states in semiconductors in a subversive way, which can co‐utilize the atomic electric field and built‐in electric field to harvest energy from the mechanical movements, especially for achieving an ultrahigh current density power source.

## Graphical abstract

Surface states are systematically revealed to enhance the performance of a dynamic Schottky direct current generator, which can generate an ultrahigh current density of 2.7 × 10^5^ A m^−2^ and power density of 1262.0 W m^−2^ by co‐utilizing the built‐in electric field and atomic electric field, making it more suitable for many applications especially in small area power collection.

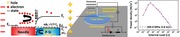

Nowadays, many different kinds of novel generators have been introduced to convert nature power into electrical power, such as mechanical energy,^1^, ^2^ hydraulic energy,^3^ solar energy,^4^, ^5^ thermal energy,^6^ etc. These generators all possess an electric field to output power, however, the large electric field induced by Schottky diode has been rarely explored to generate power.^1^, ^7^ Schottky diode has been widely used in electronic industry, such as high‐power and high‐speed integrated circuits, since its invention in the 1940s.^8^ However, impacted by thought inertia, people always use Schottky diode in static structures, limiting applications derived from Schottky diode, and researches for dynamic Schottky diode have been rarely explored.^1^ Recently, we have demonstrated the dynamic Schottky diode based direct‐current generator, which exhibited its unique and fascinating physical picture, providing one efficient way to convert mechanical energy into electrical energy.^1^, ^9^ However, the utilization of the novel physical mechanism behind the dynamic Schottky diode still needs to be further investigated for boosting up the performance of dynamic Schottky generator.

Here, we demonstrate a dynamic Schottky generator by horizontally sliding Fe needles on silicon with power density of 1262.0 W m^−2^ that even above the sunlight power density incident on the earth.^4^ In static Schottky barrier, surface states in semiconductors have been frequently reported to exert influence on the contact properties and performance of electronic devices.^10^ These surface states are caused by dangling bonds or vacancies, which capture the electrons or holes that flow through the interface, and is detrimental to the performance of the static Schottky barrier devices. However, the striking characteristic of dynamic Schottky barrier is that some parts of the electrons and holes need not cross over the interface between metal and semiconductor, which is entirely distinct from the static Schottky diode theory. Moreover, the carriers can be rebounded back by charged surface states which act as the rebounding centers. The moving directions of these carriers will be switched by the built‐in electric field and accelerated by both the charged surface states and built‐in electric field. Through increasing the surface states of the interface, we demonstrate direct‐current generator with an ultrahigh current density of 2.7 × 10^5^ A m^−2^ by co‐utilizing the built‐in electric field and atomic electric field, which is orders of magnitude higher than solar cells and other macroscopical mechanical energy‐electrical energy conversion generator.^1^, ^7^, ^9^, ^11^ This direct‐current dynamic Schottky diode based generator has a vast potential in the future development of various fields, such as self‐driven and wearable devices, artificial intelligence field, industrial analog signal transmission, etc.^1^

We set up our experiment that contains a frame, a motor, an actuator, a piezometer with a metal needle, a slide rail, a pressure controller unit, and a silicon wafer (Figure S1, Supporting Information). **Figure** **1**a shows the equivalent schematic diagram of the circuit and the dynamic charging process of the generator. The work function of our p‐type silicon is 5.14–5.19 eV, and Fe needle is 4.67–4.81 eV, respectively.^12^ Due to the work function difference between silicon and Fe needle, a built‐in electric field is formed. We propose the power output of this generator as a result of the balance breakdown between diffusing current and drift current, here we define drift current as *I*_D_ and diffusing current as *I*_E_ as shown in Equations (1) and (2)

(1) $$I_{D}=qp\mu_{p}E$$

(2) $$I_{E}=-qD_{p}\nabla p$$

where *µ*_p_ is the hole mobility, *D*_p_ is the hole diffusion coefficient in semiconductor, *q* is the elementary charge, *p* is the position‐dependent hole density in semiconductor, *E* is the built‐in electric field, respectively. In detail, as the needle moves on the surface of silicon, the needle tips and silicon establish the Schottky contact, while the needle ends and silicon lose the Schottky contact, leading to the balance breakdown of *I*_D_ and *I*_E_, some of the diffusing electrons and holes around the dynamic Schottky barrier have no way to cross over the depletion layer as shown in Figure 1b. Thus, the electrons and holes will be rebounded back by the built‐in field naturally, which is the origin of electricity output.

**Figure 1:**
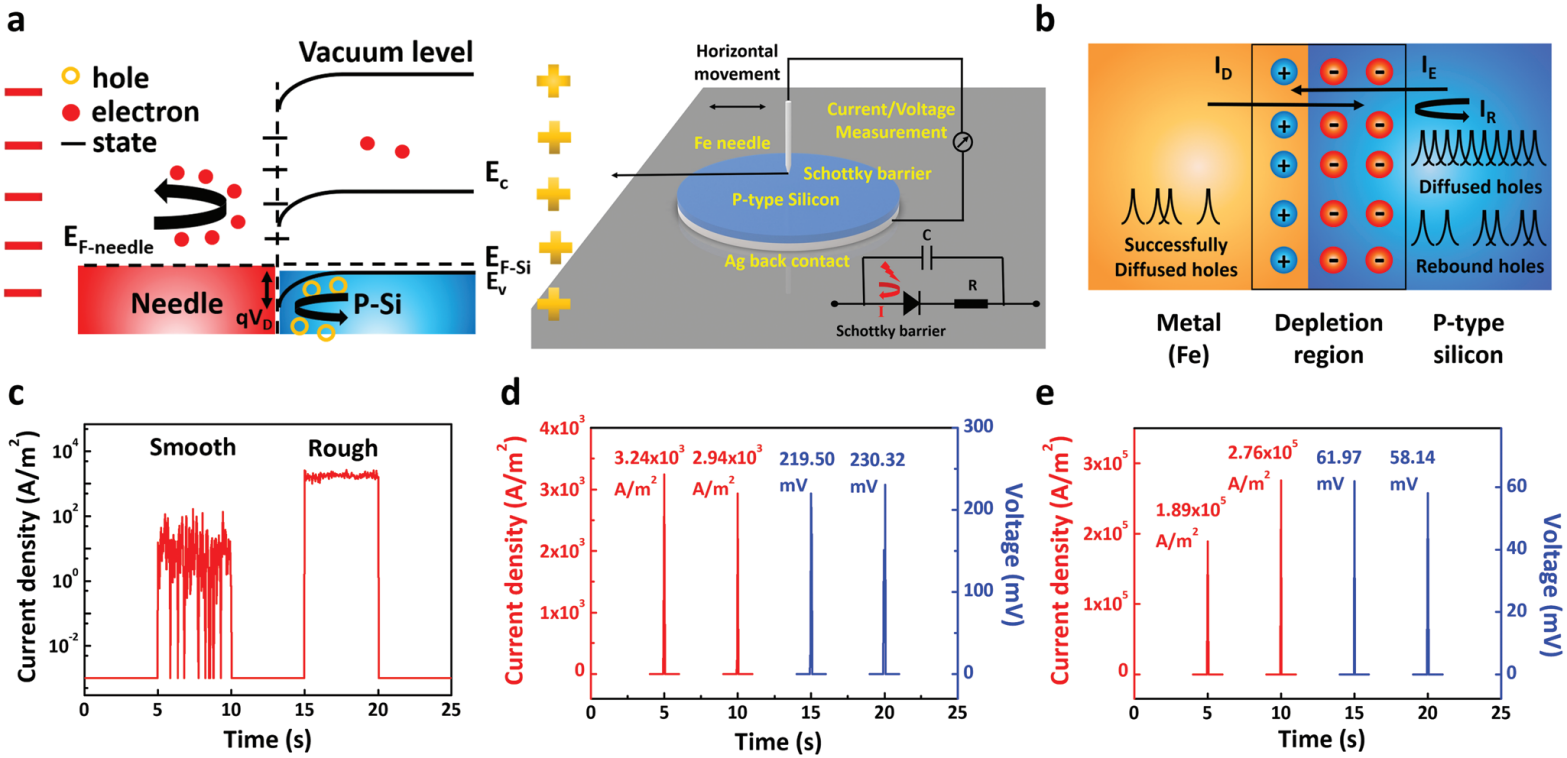
Schematic diagram of external/internal structure of dynamic Schottky generator and peak short circuit power output of dynamic Fe/silicon Schottky generator. a) The schematic diagram of the dynamic Schottky generator, the diagram of the equivalent circuit inside the dynamic Schottky generator and the dynamic charging process of the generator. b) *I*_D_, *I*_R_, and *I*_E_ inside the dynamic Schottky generator. *I*_R_ is the result of the balance breakdown between *I*_D_ and *I*_E_. c) Current output at pressure of 50.0 MPa and velocity of 0.2 m s^−1^ when needle is sliding on smooth silicon surface and rough silicon surface, here we circle the needle on silicon aiming to get more stable direct current output. d) The peak current density and voltage output for dynamic Fe/smooth silicon Schottky generator with the velocity of 0.8 m s^−1^ and pressure of 200.0 MPa. e) The peak current density and voltage output for dynamic Fe/rough silicon Schottky generator at velocity of 0.8 m s^−1^ and pressure of 200.0 MPa. All the contact area between Fe and silicon here is 0.1 mm^2^, all the errors of pressure here are ±10.0 MPa.

There are many scratches and defects on the silicon wafers after several slides. As shown in Figure 1c, when the needle slides through the surface of rough silicon that possesses many defects, the current output increases significantly under the same condition, indicating that surface states indeed have impacts on power output. By circling the needle over rough silicon with lower accelerated velocity, we prove our generator can continuously generate direct current. The continuously generated direct current output as shown in Figure 1c and Figure S2 (Supporting Information) indicates that the generator can work without obvious charge–discharge in silicon when sliding, which is in agreement with the proposed mechanism. Speed of 0.8 m s^−1^ and pressure of 200.0 MPa is found to be the best choice for the maximum power output. Dynamic Fe/smooth silicon Schottky generator can output a peak current of 0.32 mA and a peak voltage of 230.39 mV, while dynamic Fe/rough silicon Schottky generator can output a peak current of 27.56 mA and a peak voltage of 61.97 mV, as shown in Figure 1d,e, respectively. Herein, the current density can reach a peak that higher than 3.2 × 10^3^ and 2.7 × 10^5^ A m^−2^ for the dynamic Fe/smooth silicon and Fe/rough silicon Schottky generator, respectively (all the contact area between Fe and silicon here is 0.1 mm^2^). For moving rough silicon over Fe film, we also observed similar high current density as shown in Figure S6 (Supporting Information).

We indicate that surface states have significant impacts on the power output of our generator, as shown in **Figure** **2**. The surface states can act as the rebounding centers and enhance the current output density that is totally different from the case of static Schottky diode. The increased defects on the rough surface weak the Schottky barrier between metal and silicon, and the Fermi level of silicon is pinned in the bandgap of silicon, and result in lower voltage output irrelated with Fermi level difference between metals and silicon. Figure 2a shows the current–voltage characteristics between Fe needle and rough/smooth silicon, where the red line represents the current–voltage characteristics of Fe/smooth silicon Schottky barrier and blue line represents the current–voltage characteristics of Fe/rough silicon Schottky barrier. The rectified Schottky barrier between Fe and rough silicon is obviously weaker than the smooth one, which is in line with the abovementioned mechanism. However, these increased surface states can act as the rebounding centers locating in the space charge region where has the built‐in field, thus these surface states increase the chances of carriers being rebounded back by both built‐in field and atomic electric field, adding the net current output. Figure 2b,c shows the energy diagram between needle and rough silicon as the surface states density of rough silicon increases significantly compared to Figure 1a. Scanning electron microscope (SEM) images of different silicon types are shown in Figure S3 (Supporting Information). To investigate the dependence of the metals, we select six types of needle materials (Fe, Au, Ag, Cu, Al, and Pt) to slide the needles on the surface of smooth/rough p‐type silicon. Data shown in Figure 2d and Figure S4 (Supporting Information) demonstrates that all kinds of needles get several orders of magnitude higher current density output when sliding on rough silicon, proving these defects provide more rebounding or reflecting centers on the surface. They all generate almost the same low voltage beyond 100 mV, as shown in Figure S4 (Supporting Information), which demonstrates the Fermi level pinning effect of silicon. The high definition XPS spectra result for Si is showed in Figure 2e. There are two Si 2p peaks for rough silicon locating at 98.88 and 102.98 eV, which shift to lower energy compared with the Si 2p peak of intrinsic Si and SiO_2_, respectively,^13^ indicating there are dangling bonds of Si—O and Si—Si on the rough silicon surface, which act as the rebounding centers in the dynamic Schottky generator. The dangling bonds of Si—O and Si—Si are formed by the destruction of O—Si—O and Si—Si bonds, and can be formed by partial oxidation of Si surface in air.

**Figure 2:**
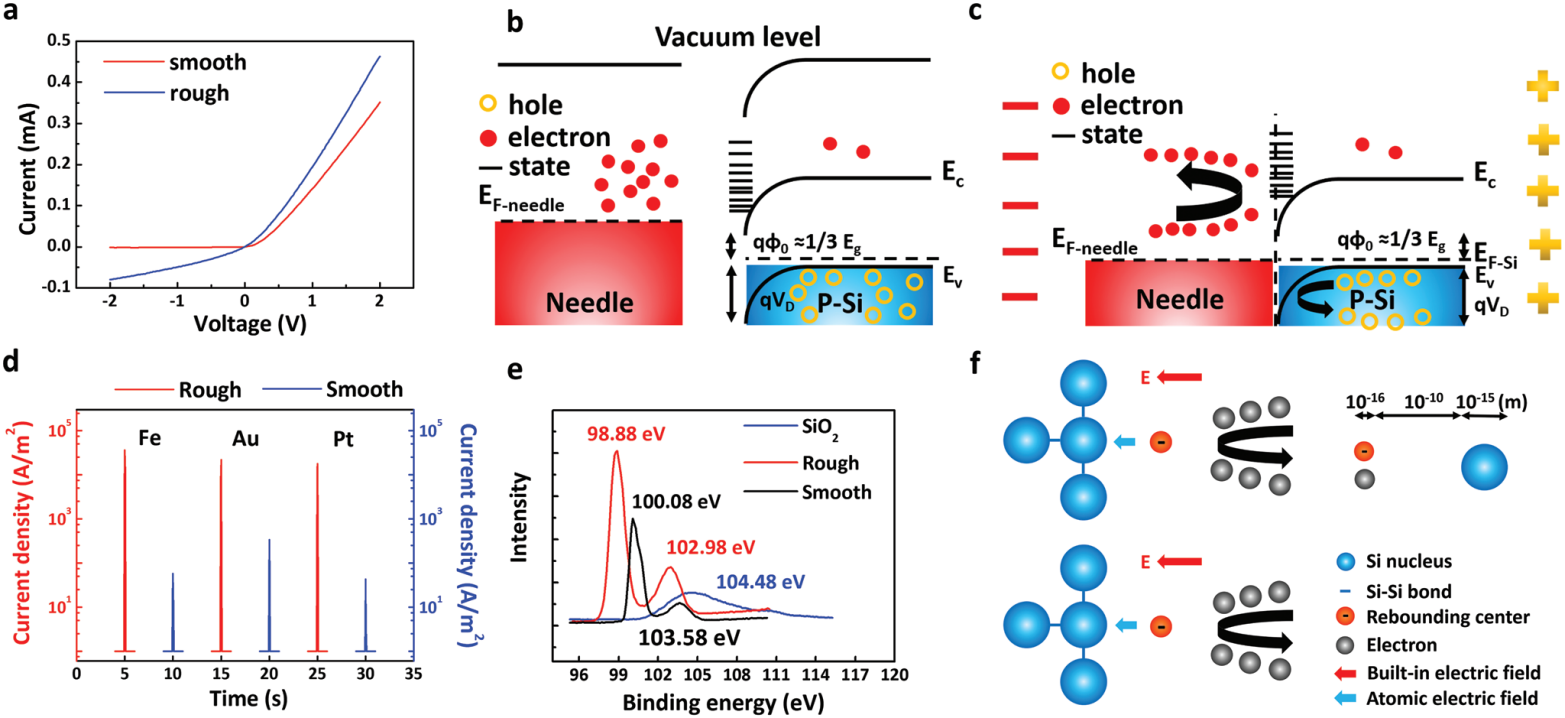
Current output can be enhanced by more surface states acting as the rebounding centers. a) Current–voltage curves between Fe needle and smooth/rough silicon, the leakage current of Fe/rough silicon Schottky barrier is larger. b) Energy band diagram of rough silicon/needle Schottky generator before Schottky barrier forms, there is a potential barrier on the silicon surface due to the Fermi level pinning effect under high surface states. c) Energy band diagram for rough silicon/needle inside the generator, more carriers are rebounded back due to high surface states, the potential between metal needle and silicon is decided by the potential barrier on the silicon surface rather than the Fermi level difference between metal and silicon due to the Fermi level pinning effect. d) The current output of smooth/rough silicon wafers and different metals at speed of 0.2 m s^−1^ and pressure of 50.0 MPa. Current outputs of all the metals/rough silicon generator tested here are enhanced. e) High resolution Si spectra measured by XPS, the Si 2p peaks indicate there are many dangling bonds on the silicon surface. f) Schematic diagram for the reflecting process of scattering electrons. Here we select Si—Si bond as an example. As the Si—Si bonds break, more dangling bonds on the surface become the rebounding centers for free carriers, and the large electric field induced by nucleus and electron clouds also accelerate free carriers. All the errors of pressure here are ±10.0 MPa.

In contrast to the static Schottky diode, the dangling bonds can enhance the current output in the dynamic Schottky diode, which is quite inspiring. As the metal moves on the surface of the semiconductor, the diffusing carriers lose the pathway of crossing the otherwise existed Schottky contact, thus have to be reflected by the both atomic electric field and built‐in electric field. Those reflected carriers break the balance between diffusing and drift current, leading to the electricity output. This physical mechanism is self‐consistent and can explain the current and voltage output. Figure 2f shows the schematic diagram of the reflecting process of electrons accelerated and rebounded by both atomic electric field and built‐in electric field in the dynamic Schottky generator, here Si—Si dangling bond is used for an example. According to our mechanism, carriers can co‐utilize the electric field inside the atoms apart from being directly rebounded back by rebounding centers as long as the atomic electric field between nucleus and electron cloud possesses a vector component in the same direction as the built‐in electric field. Besides, the width of built‐in electric field is orders of magnitude larger than atomic field, the carriers have little possibility to reach the Si atoms, which possess dangling bonds before being reflected by built‐in electric field. Thus, a higher performance dynamic Schottky generator device structure can be designed by adopting the reflecting or rebounding back carrier model.

In addition to surface states, the current and voltage output can be further improved by other methods like increasing pressure, velocity, resistivity of silicon, etc. **Figure** **3**a,b shows the dependence of power output on pressure and velocity. As shown in Figure 3a,b, current output can be enhanced by higher velocity. Here we indicate that higher velocity means more free carriers, and these carriers will be rebounded back at the same time. Current output can also be enhanced by higher pressure, then more free carriers are rebounded back. However, voltage is irrelevant with velocity or pressure as shown in Figure 3a,b. We indicate that due to the Fermi level pinning effect of silicon under high surface states, the built‐in electric field intensity and width are constant as the surface potential on silicon is constant, and the voltage output is decided by the built‐in electric field intensity and width. Figure 2d and Figure S4 (Supporting Information) also demonstrate the pinning effect inside our dynamic Schottky generator as there is no significant difference voltage output of different metals (work function varying from ≈4.2 to ≈5.2 eV)/rough silicon. As voltage output still needs further improvement, here we put two and three basic devices in series (under the velocity of 0.2 m s^−1^ and pressure of 50.0 MPa), the voltage output is shown in Figure 3c. The voltage output is basically consistent with the law of series, which demonstrates that dynamic Schottky generator can output a higher voltage by connecting many basic generator units in series. Another way to improve voltage is by increasing resistivity of silicon, five types of silicon (25.5–40/1–2/0.01–0.02/0.001–0.005/0.0002–0.001 Ω cm^−1^ from the highest to the lowest) is selected here. As the fitting curve in Figure 3d shows that voltage output increases when resistivity of silicon increases, and current density shares an opposite tendency. The current density output can be enhanced two orders of magnitude higher by decreasing the resistivity of silicon from 40 to 0.001 Ω cm^−1^ (1.3 × 10^2^, 2.6 × 10^2^, 5.3 × 10^3^, 3.6 × 10^4^, and 7.8 × 10^4^ A m^−2^ from the highest to the lowest resistivity, the range of error bar is 10%), and the voltage output can be enhanced one order of magnitude higher by increasing the resistivity from 0.001 to 40 Ω cm^−1^ (0.5, 2.1, 40.8, 39.7, and 48.4 mV from the lowest to the highest resistivity, The range of error bar is 10%). Dynamic Fe/smooth silicon of highest resistivity (25.5–40 Ω cm^−1^) Schottky generator can generate a peak voltage of 1.8 V and a current density output of 46.0 A m^−2^ (Figure S5, Supporting Information).

**Figure 3:**
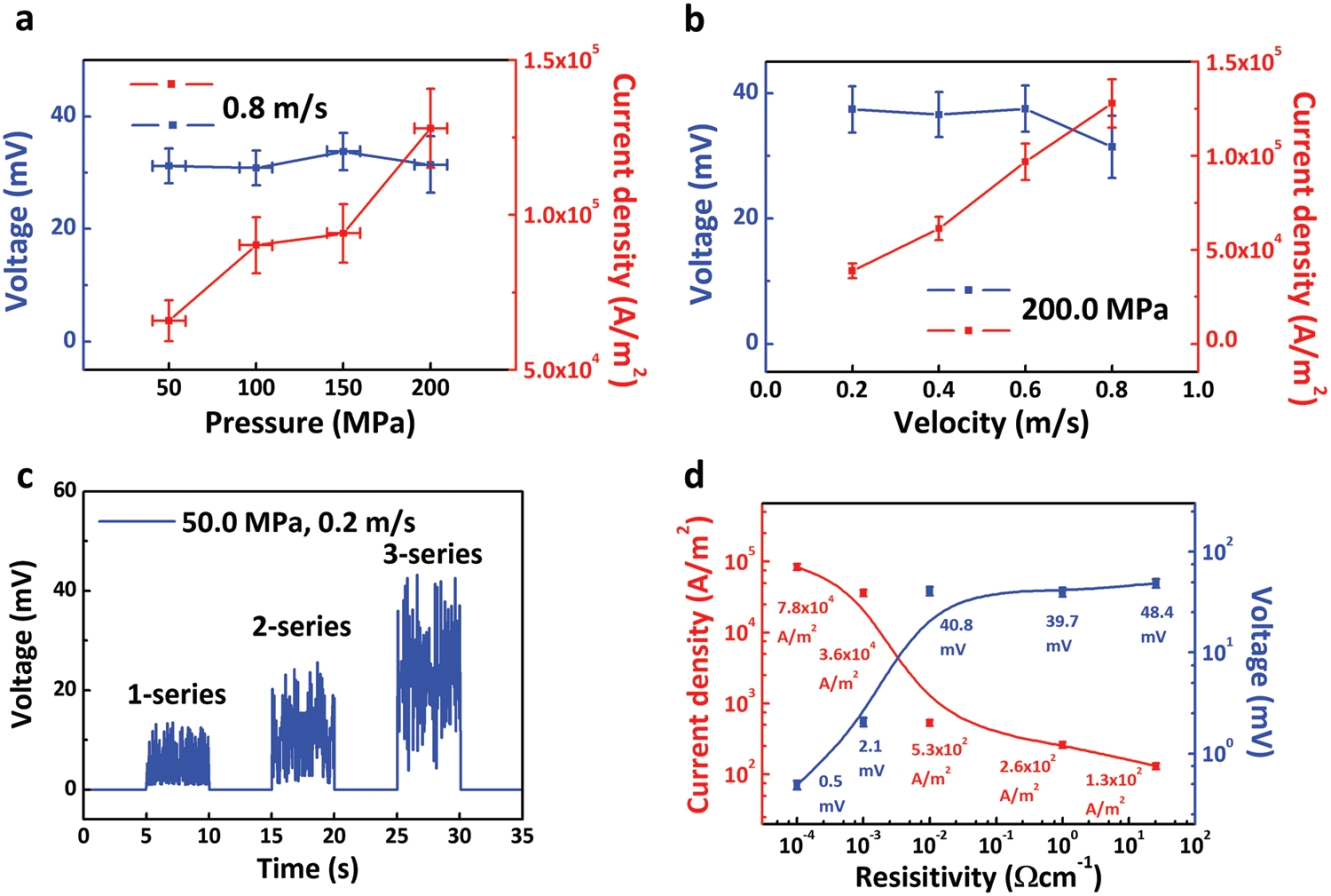
Factors on the power output of dynamic Fe/rough silicon Schottky generator. a) The dependence of voltage and current density output on pressure at velocity of 0.8 m s^−1^. b) The dependence of voltage and current density output on velocity at pressure of 200.0 MPa. c) The voltage output of two and three dynamic Fe/rough silicon Schottky generator units after we connect them in series at pressure of 50.0 MPa and velocity of 0.2 m s^−1^. d) The current and voltage output of Fe/rough p‐type silicon of different resistivities at velocity of 0.2 m s^−1^ and pressure of 50.0 MPa. All the contact area between Fe and silicon here is 0.1 mm^2^, all the errors of pressure here are ±10.0 MPa.

As our dynamic Schottky generator has reached ultrahigh current density (2.7 × 10^5^ A m^−2^), many promising applications like wearable devices, analog signal transmission will be discovered. Besides high current density, power density output of dynamic Schottky generator is also essential to evaluate the performance of generator. Herein, we select several loads of resistance from 0.1 Ω to 47.0 KΩ, current density and voltage output of dynamic Fe/rough silicon Schottky generators are shown in **Figure** **4**a. As the fitting curve shown in Figure 4b, when the load resistance is around 3.0 Ω, our generator reaches the power density peak of 1262.0 W m^−2^, which is considerably high for mechanical energy‐electrical energy conversion generator. The efficiency of our generator become more vital as we have demonstrated that our dynamic Schottky generator possesses great potential to output high power density. Here, the efficiency η is defined as

(3) $$\eta =\frac{P_{\mathrm{out}}}{P_{\mathrm{in}}}=\frac{V_{\mathrm{out}}\times I_{\mathrm{out}}}{f\times v}$$

*V*_out_ represents output voltage on external load, *I*_out_ represents output current on external load, *f* represents the frictional force and *v* represents the velocity. Dependence of power density and efficiency on velocity and pressure is shown in Figure 4c,d, from which we find that power density output is irrelevant with pressure and velocity, respectively, and efficiency is negatively correlated with pressure and velocity. We indicate when the pressure and velocity is over limit, the further increased power input cannot be efficiently transferred to electricity, which means that the rebounding centers can be gradually saturated under one certain pressure and velocity, besides, there is more recombination of carriers which establish the built‐in electric field under higher velocity, leading to the reduction of efficiency from 0.84% to 0.19%. To achieve practical applications, the efficiency of our generator still needs further improvement, especially under high input power condition. As higher current and power density output enhanced by surface states is reported for the first time, the efficiency of dynamic Schottky generator can be further improved by optimizing the structure and circuit, such as a sophisticated design of atomic electronic rebounding center for increasing the rebounding back possibility for single rebounding center, etc.

**Figure 4:**
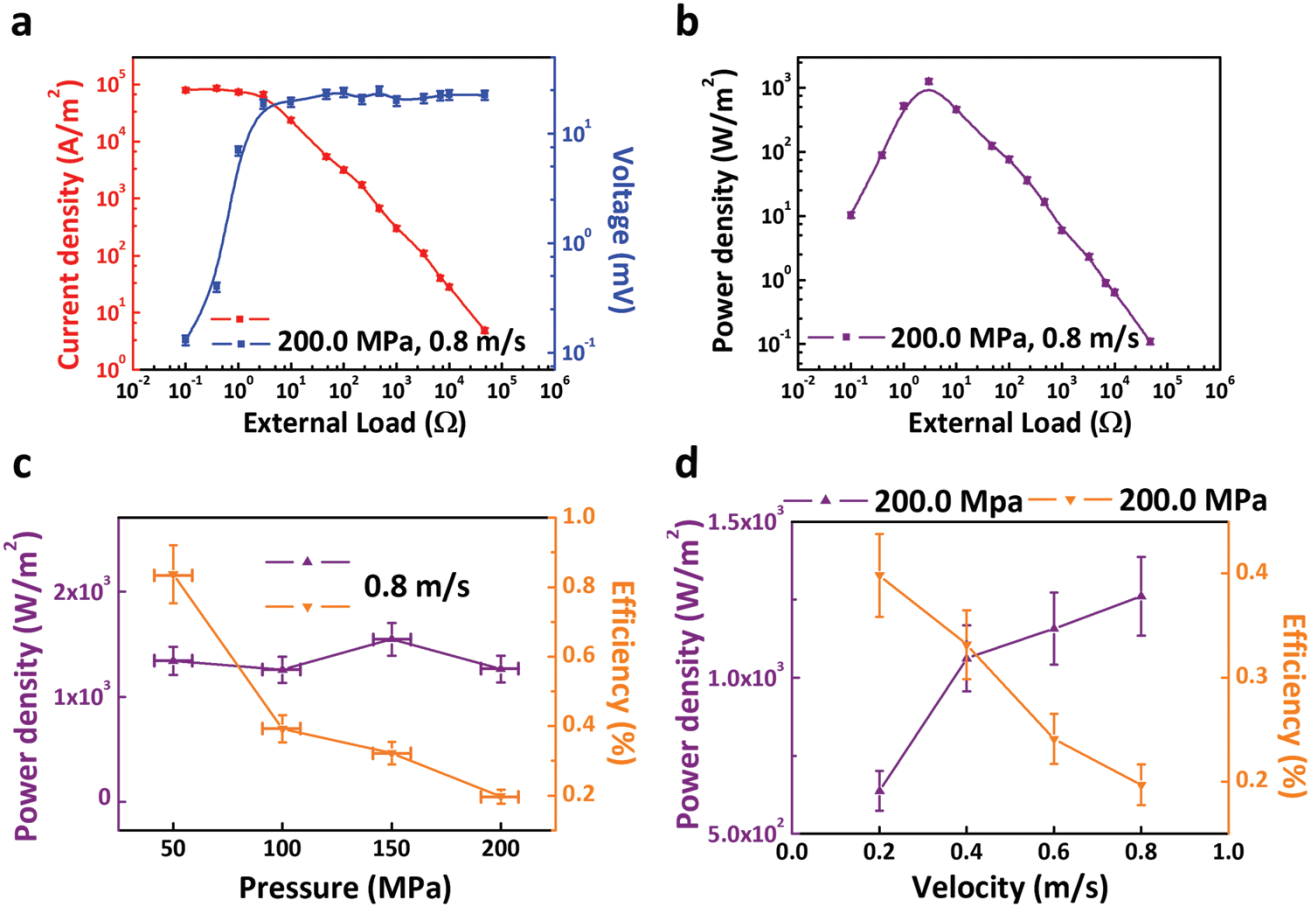
Power density and efficiency of dynamic Fe/rough silicon Schottky generator. a) The current and voltage output on different load resistances at velocity of 0.8 m s^−1^ and pressure of 200.0 MPa. b) The power density output on different load resistances at the velocity of 0.8 m s^−1^ and pressure of 200.0 MPa, the power density output peak is reached on the load resistance of 3.0 Ω. c) The dependence of power density output and efficiency on pressure at velocity of 0.8 m s^−1^. d) The dependence of power density output and efficiency on velocity at pressure of 200.0 MPa. All the contact area between Fe and silicon here is 0.1 mm^2^, all the errors of pressure here are ±10.0 MPa.

To summarize, we have demonstrated a dynamic Schottky generator which can output ultrahigh current and power density by co‐utilizing the built‐in electric field and atomic electric field. Differed from many other macroscopic generators that convert mechanical energy into electrical energy, our dynamic Schottky generator can output ultrahigh direct current and power density without any complex external circuits. Power output is generated by the built‐in electric field separation of free carriers from metal and silicon and enhanced by atomic electric field induced by surface states on silicon surface. These states can act as the rebounding and accelerating centers that located in the space charge region where has the built‐in field according to our rebounding back mechanism, making it a revolutionary research for exploring beneficial effects induced by surface states rather than side effects in Schottky diode. As an ultrahigh power density output of 1262.0 W m^−2^ and an ultrahigh current density output of 2.7 × 10^5^ A m^−2^ reached by dynamic Fe/rough silicon Schottky generator, many promising future applications like microscopic energy harvesting system, analog signal transmission, artificial intelligence field, etc. Moreover, we can further improve our power output and efficiency by optimizing the circuit, interface of Schottky barrier, rebounding centers, velocity, etc.

## Experimental Section

*Devices Fabrication*: All kinds of needles were dipped into 10 wt% HCl for 10 min to remove the native oxide layer on the surface and also washed in deionized water. Similarly, the single side polished p‐type Si substrate was dipped into 10 wt% HF for 10 min to remove the native oxide layer in the interface, and then washed in deionized water. Then, Ag (100 nm) electrode was fabricated by annealing on the back side of silicon wafer. The needles were pressed closely on the p‐type Si substrate by the equipment, making sure a solid electrical contact between metal needles/p‐type Si substrate can be achieved. The silicon wafers with silver ohmic contact have been used in the experiments, which have below detailed properties unless otherwise stated: 525 ± 10 µm, orientation <100>, resistivity: 0.001–0.005 Ω cm^−1^. The rough silicon face is achieved by scratching with abrasive paper.

*Physical Characterization Methods*: The microscopic image of the generator was characterized with ZEISS optical microscopy. The current–voltage (*I*–*V*) curve of the Schottky barrier was measured by Keithley 2400 system. The real‐time voltage and current output were recorded in real time by a Keithley 2010 system, which was controlled by a Labview‐based data acquisition system with a sampling rate of 25 s^−1^. The Keithley 6485 pico‐ammeter was also used to verify the accuracy of current output, which was controlled by a LabView‐based data acquisition system with a sampling rate of 100 s^−1^. The force was measured by a pressure meter with precision to one decimal place.

*Materials Characterization Analysis*: The XPS experiments equipment here is Thermo Scientific ESCALAB 250Xi, the light source is Al Ka 1486.6 eV, and the standard peak for C is 284.8 eV. SEM was measured by HITACHI S‐4800 system. The absorption spectrum was measured by Agilent Carry 7000 UV–vis spectrometer.

## Conflict of Interest

The authors declare no conflict of interest.

## Supporting information

Supplementary
