# Supplementary material for: Surface States Enhanced Dynamic Schottky Diode Generator with Extremely High Power Density Over 1000 W m−2
Source: Adv Sci (Weinh). 2019 Oct 25;6(24):1901925. doi: 10.1002/advs.201901925 (PMC6918112; doi:10.1002/advs.201901925)
Supplement: Supplementary file 1 — Supplementary [file ADVS-6-1901925-s001.pdf]

Copyright WILEY-VCH Verlag GmbH & Co. KGaA, 69469 Weinheim, Germany, 2019.

ADVANCED SCIENCE
Open Access

Supporting Information

for *Adv. Sci.,* DOI: 10.1002/advs.201901925

Surface States Enhanced Dynamic Schottky Diode Generator with Extremely High Power Density Over 1000 W m^{-2}

Shisheng Lin, Runjiang Shen, Tianyi Yao, Yanghua Lu, Sirui Feng, Zhenzhen Hao, Haonan Zheng, Yanfei Yan, and Erping Li*

Article type: Communication

Surface states enhanced dynamic Schottky diode generator with extremely high power density over 1000 W/m^{-2}

Shisheng Lin, Runjiang Shen, Tianyi Yao, Yanghua Lu, Sirui Feng, Zhenzhen Hao, Haonan Zheng, Yanfei Yan and Erping Li*

Prof. S. -S. Lin, R. -J. Shen, T. -Y. Yao, Y. -H. Lu, S. -R. Feng, Z. -Z. Hao, H. -N. Zheng, Y. -F. Yan

College of Microelectronics, College of Information Science and Electronic Engineering, Zhejiang University, Hangzhou, 310027, China

Prof. S. -S. Lin

State Key Laboratory of Modern Optical Instrumentation, Zhejiang University, Hangzhou, 310027, China

Prof. E. -P Li

Key Laboratory of Micro-Nano Electronics and Smart System of Zhejiang Province, College of Information Science and Electronic Engineering, Zhejiang University, Hangzhou 310027, China.

E-mail: shishenglin@zju.edu.cn.

Keywords: (dynamic Schottky diode, direct current generator, high power density, high current density, surface states, rebounding centers)

Supplementary information:

Figs. S1 to S6

1. Equipment for experiments
2. Direct power output of dynamic Schottky generator
3. Scanning electron microscope (SEM) images of different silicon wafers
4. Power output of different materials and supplementary data to further clarify the influence of surface states
5. Power output of Fe/smooth silicon with high resistivity
6. Current output for silicon sliding on Fe film

Supplementary Figure:

1. Figure S1

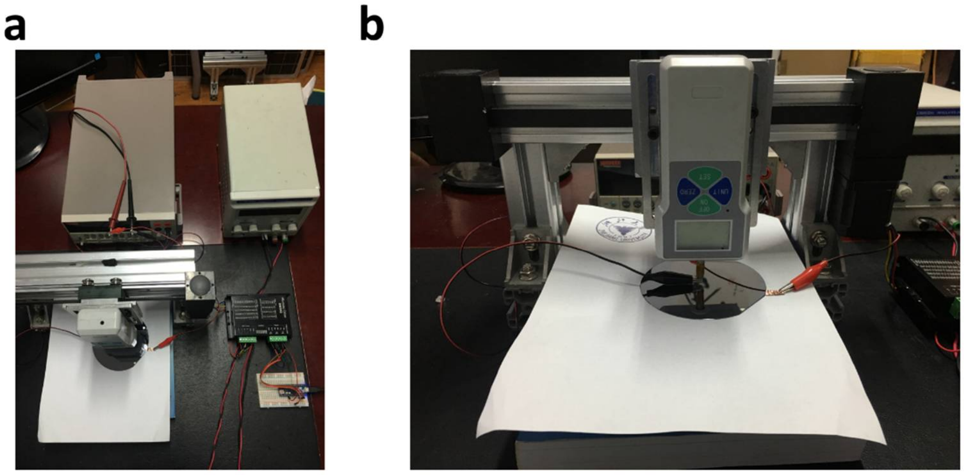

Figure S1: (a) Equipment for our experiments. (b) Closer image for our equipment.

Here we show our equipment for experiments. We use a computer to send Arduino program to Microcontroller Unit (MCU) and charge MCU, then MCU will control the motor which is charged by a common voltage-stabilized source. We can change velocity by computer and change pressure by adjusting height of the piezometer. Voltage and current output are measured by Keithley 2010 system. When we start the motor, the needle bounded on the piezometer will slide over the surface of the silicon wafer along the rail. The screw applies a fixed force on the piezometer, which ensures the controlment of both speed and pressure.

2. Figure S2

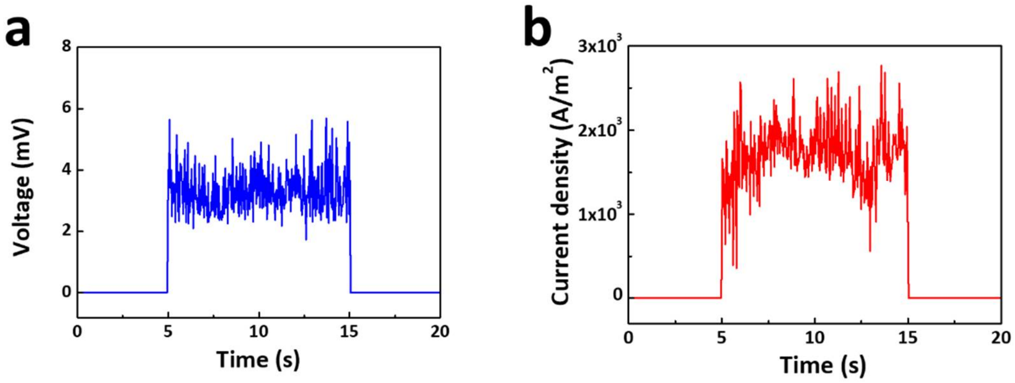

Figure S2: (a) Voltage output when the needle is circling around the rough silicon surface at pressure of 50.0 MPa and velocity around 0.2 m/s. (b) Current output when needle is circling around the silicon surface at pressure of 50.0 MPa and velocity around 0.2 m/s.

In order to prove that the direct current is output by our generator, here we show power output of circling movement of needles which possesses lower acceleration. We can see that the power output of circling movement is more stable than that of rectilinear motion, demonstrating the direct current is indeed generated.

3. Figure S3

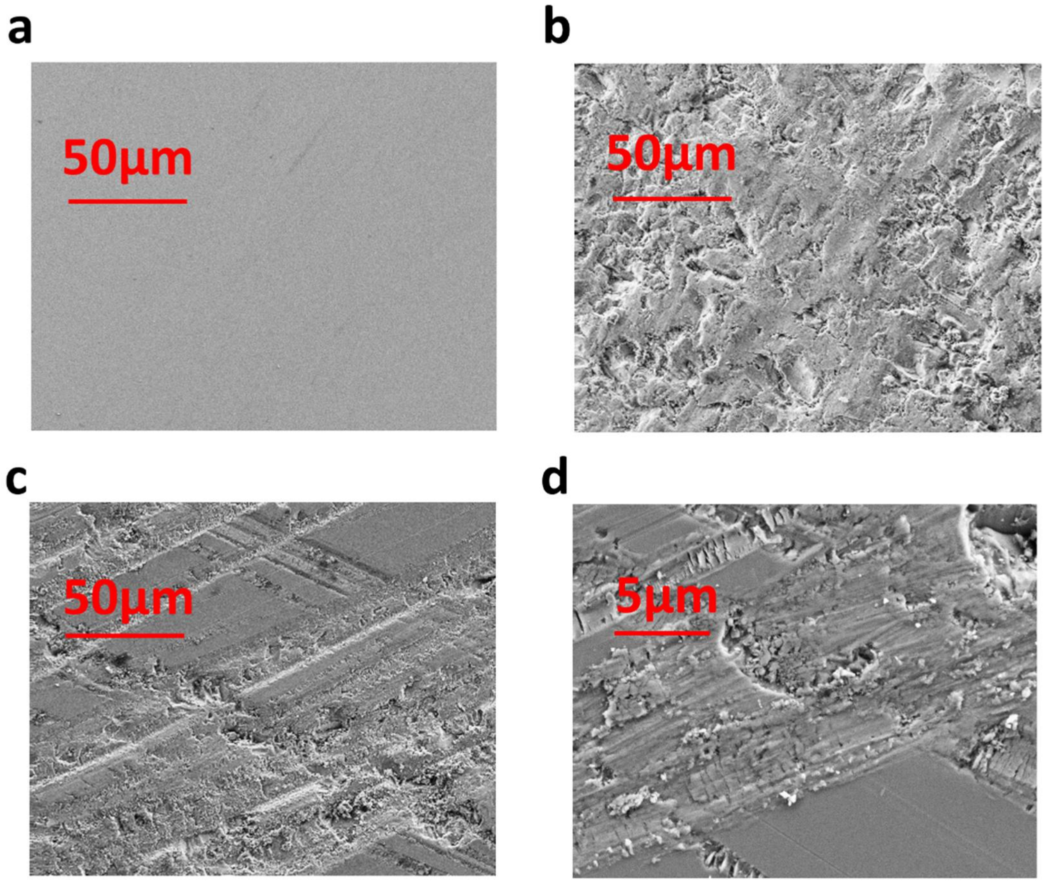

Figure S3: SEM images of different silicon wafers (a) A smooth silicon wafer after being slid (b) A rough silicon wafer after being polished and cleaned. (c) A rough silicon wafer after being polished without being cleaned ethyl alcohol. (d) A 10 times zoom-in image of one section in c.

Supplementary Figure S3a is the 500x image of smooth silicon wafer without needle's sliding. Supplementary Figure S3b is the 500x image of polished silicon (polished by abrasive paper) after being cleaned by ethyl alcohol. Supplementary Figure S3c is the 500x image of polished silicon without being cleaned by ethyl alcohol. Obviously, there are many scratches unevenly on the surface of silicon. More

details can be seen in Supplementary Figure S3d, which is a 5000x image of the same silicon surface.

4. Figure S4

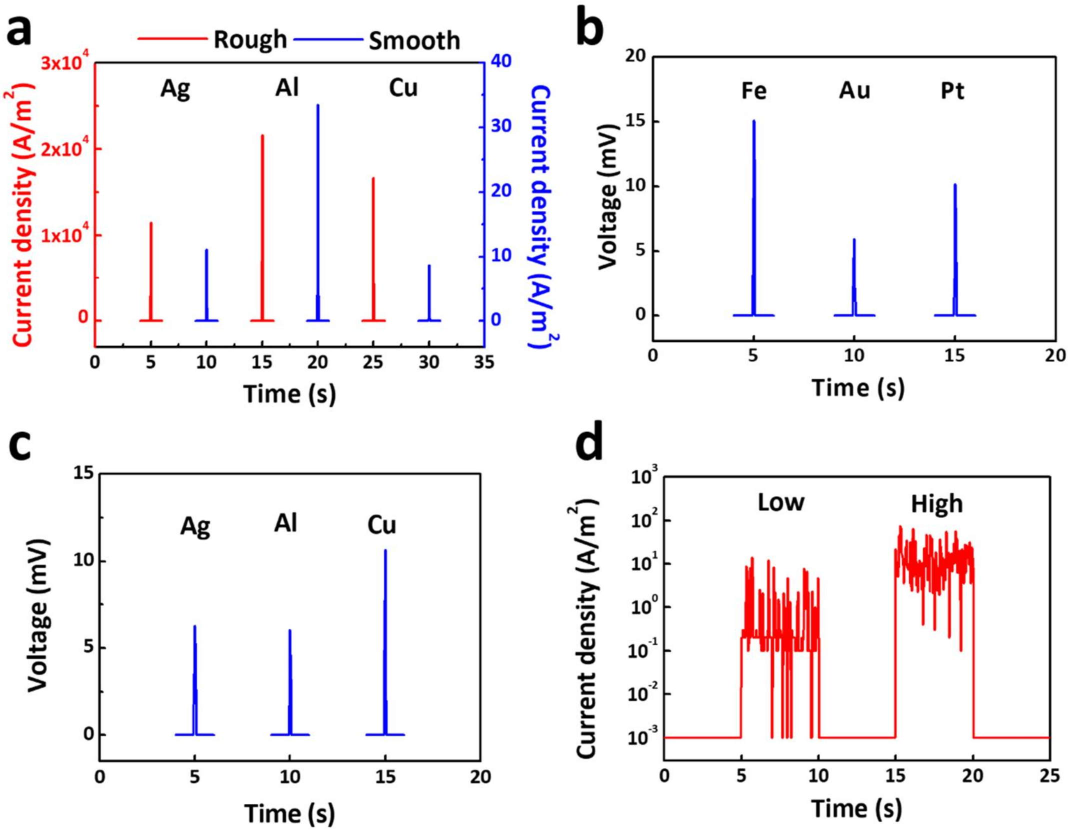

Figure S4: (a) Supplemental data for the current output between smooth/rough silicon wafers and Ag/Al/Cu at speed of 0.2 m/s and pressure of 50.0 MPa. (b) Supplementary data for the voltage output between rough silicon wafers and Fe/Au/Pt at speed of 0.2 m/s and pressure of 50.0 MPa. (c) Supplementary data for the voltage output between rough silicon wafers and Ag/Al/Cu at speed of 0.2m/s and pressure of 50.0 MPa. (d) Supplementary data for the current output between GaAs wafers which possess low/high surface states and Fe at speed of 0.2 m/s and pressure of 50.0 MPsa.

Here we show some supplemental data for current/voltage output for needles made of different materials. We can see that current output between these materials (Ag, Al, Cu) and silicon shares the same tendency with Fe, Au and Pt. However, all kinds of materials/silicon Schottky generator outputs lower voltage beyond 100 mV

due to lower Fermi level difference caused by higher surface states as shown in Figure S4b and S4c. Figure S4d shows current output of GaAs/Fe Schottky generators which possess high/low surface states. Here passivation of GaAs within the active area was realized by remote NH_3 plasma treatment for 5 min with 120 W 27.5 MHz RF generator.[1]

5. Figure S5

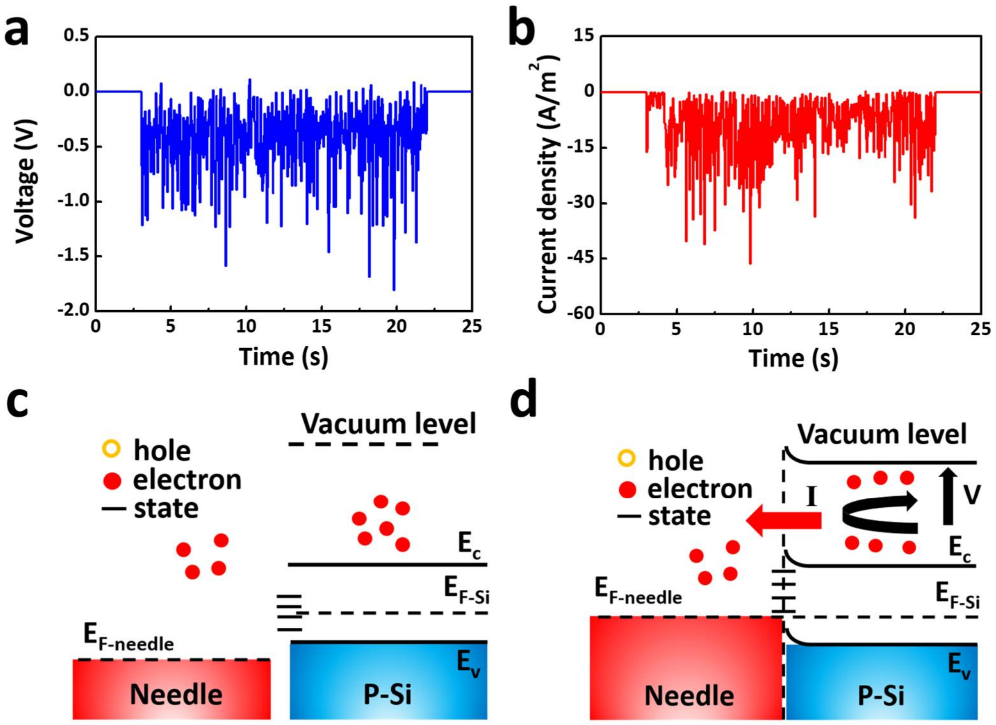

Figure S5: (a) The voltage output between smooth silicon with high resistivity /Fe needle at pressure of 200.0 MPa and velocity of 0.8 m/s. (b) The current output between smooth silicon with high resistivity/Fe needle at pressure of 200.0 MPa and velocity of 0.8 m/s. (c) The energy band diagram of two materials (Fe/silicon with high resistivity). (d) The energy band diagram of the Schottky barrier inside the generator.

Here we use p-type silicon with a resistivity of 25.5 - 42.5 Ωm^{-1} (baron doped, orientation <100>, resistivity: 0.001 - 0.005 $\Omega \cdot cm^{-1}$, argentum back contact, no bias voltage), which means it possesses Fermi level of 4.72 – 4.75 eV under ideal conditions. The voltage output here is 1 order of magnitude higher than that silicon of 0.001- 0.005 $\Omega \cdot cm^{-1}$/Fe Schottky generator, however, it gets 2 orders of magnitude

lower current output which makes the power output lower than previous one. It is interesting to note that it has opposite power direction with silicon of 0.001 - 0.005 $\Omega\cdot cm^{-1}$. Energy band diagram is shown in c and d, as the built-in electric field gets opposite direction, the power output shares the same direction. As work function is dependent on the cleanliness degree of the surface,[2] We infer that the work function of silicon increases after being polished, then rough silicon of 25.5 - 42.5 $\Omega\cdot cm^{-1}$/Fe Schottky generator outputs forward current.

5. Figure S6

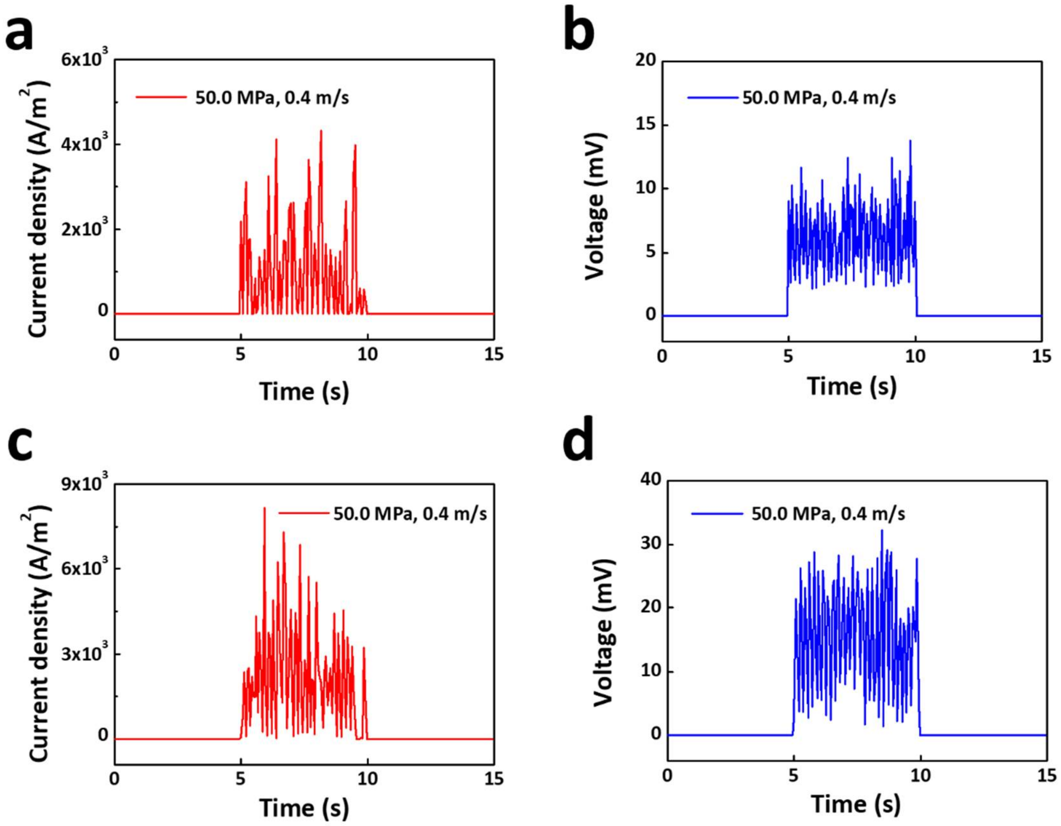

Figure S6. a) Current output for silicon sliding on Fe film. b) Voltage output for silicon sliding on Fe film. c) Current output for Fe needle sliding on silicon. d) Voltage output for Fe needle sliding on silicon.

All the experiments here are under the velocity of 0.4 m/s and pressure of 50.0 MPa (applied to silicon in (a)(b) and Fe needle in (c)(d)).

Reference

[1] X. Li, W. Chen, S. Zhang, Z. Wu, P. Wang, Z. Xu, H. Chen, W. Yin, H. Zhong, S. Lin, *Nano Energy* **2015**, *16*, 310.

[2] John R. Rumble, ed. Electron Work Function of the Crystalline Elements in CRC Handbook of Chemistry and Physics, CRC Press Taylor & Francis,LON **2018**.
